# A male patient with acromegaly and breast cancer: treating acromegaly to control tumor progression

**DOI:** 10.1186/s12885-015-1400-0

**Published:** 2015-05-12

**Authors:** Paola Leporati, Rodolfo Fonte, Luca de Martinis, Alberto Zambelli, Flavia Magri, Lorenzo Pavesi, Mario Rotondi, Luca Chiovato

**Affiliations:** 1 Unit of Internal Medicine and Endocrinology, Fondazione Salvatore Maugeri I.R.C.C.S., Laboratory for Endocrine Disruptors, Chair of Endocrinology, University of Pavia, 27100 Pavia, Italy; 2 Unit of Medical Oncology, Fondazione Salvatore Maugeri I.R.C.C.S., 27100 Pavia, Italy

**Keywords:** Acromegaly, Tamoxifen, Pegvisomant, Breast cancer

## Abstract

**Background:**

Acromegaly is a rare disease associated with an increased risk of developing cancer.

**Case presentation:**

We report the case of a 72-year-old man who was diagnosed with acromegaly (IGF-1 770 ng/ml) and breast cancer. Four years before he suffered from a colon-rectal cancer. Pituitary surgery and octreotide-LAR treatment failed to control acromegaly. Normalization of IGF-1 (97 ng/ml) was obtained with pegvisomant therapy. Four years after breast cancer surgery, 2 pulmonary metastases were detected at chest CT. The patient was started on anastrozole, but, contrary to medical advice, he stopped pegvisomant treatment (IGF-I 453 ng/ml). Four months later, chest CT revealed an increase in size of the metastatic lesion of the left lung. The patient was shifted from anastrozole to tamoxifen and was restarted on pegvisomant, with normalization of serum IGF-1 levels (90 ng/ml). Four months later, a reduction in size of the metastatic lesion of the left lung was detected by CT. Subsequent CT scans throughout a 24-month follow-up showed a further reduction in size and then a stabilization of the metastasis.

**Conclusions:**

This is the first report of a male patient with acromegaly and breast cancer. The clinical course of breast cancer was closely related to the metabolic control of acromegaly. The rapid progression of metastatic lesion was temporally related to stopping pegvisomant treatment and paralleled a rise in serum IGF-1 levels. Normalization of IGF-1 after re-starting pegvisomant impressively reduced the progression of metastatic breast lesions. Control of acromegaly is mandatory in acromegalic patients with cancer.

## Background

Acromegaly is a chronic disease caused by excessive growth hormone (GH) secretion by a GH-secreting pituitary adenoma. Acromegalic patients suffer from high morbidity and mortality mainly due to cardiovascular, metabolic and respiratory diseases [1, 2]. The risk of developing malignancies is also increased in acromegalic patients [3]. However, cancer-related mortality is increased only in acromegalic patients with poorly controlled disease, as assessed by high circulating levels of Insulin-like-Growth-Factor 1 (IGF-1) [4, 5]. Cancers of the thyroid, breast and colon-rectum are the most commonly encountered malignancies in patients with acromegaly [6–8]. Thus, screening for tumors is recommended before, during and after treatment for acromegaly [6]. Long term follow up is also mandatory, mainly in patients who, despite treatment, fail to achieve a good metabolic control, as assessed by persistently elevated serum levels of IGF-1.

Male breast cancer is a rare malignancy that accounts for 0.7 % of all breast cancers [9] and displays a significant geographic variation in its incidence [10]. The mean age at diagnosis is in the fifth decade [9] and the prognosis is generally worse than in women [11]. Several factors including obesity, chronic liver disease, genetics, and family history were reported to be implicated in the development of the disease [12].

We hereby report the case of a patient who was diagnosed with acromegaly and breast cancer. Four years before he had been successfully treated for a colon-rectal cancer. To the best of our knowledge, this is the first report of a male patient with acromegaly who developed breast cancer. In our opinion, relevant clinical and therapeutic information can be drawn from the behavior of breast cancer in relation to the metabolic control of acromegaly.

## Case presentation

A 72-years-old male patient was referred to our Unit in May 2007 for a non-toxic multinodular goiter, which had recurred following a partial thyroidectomy performed nearly 20 years before. The clinical history of the patients revealed: 1) Type two diabetes mellitus, which had been diagnosed at the age of 50 years and was treated with insulin and oral glucose-lowering drugs; 2) Hypertension since the age of 44 years, complicated by retinopathy; 3) a previous (four years before) diagnosis of colon-rectal adenocarcinoma (G2, without sub-serosal infiltration), which had been treated with surgical resection (right colectomy with ileo-colic anastomosis) and adjuvant chemotherapy. Resection margins were free of disease and surrounding lymph nodes were not involved. In the subsequent follow-up, no local relapse or metastatic spread was detected.

**Fig. 1:**
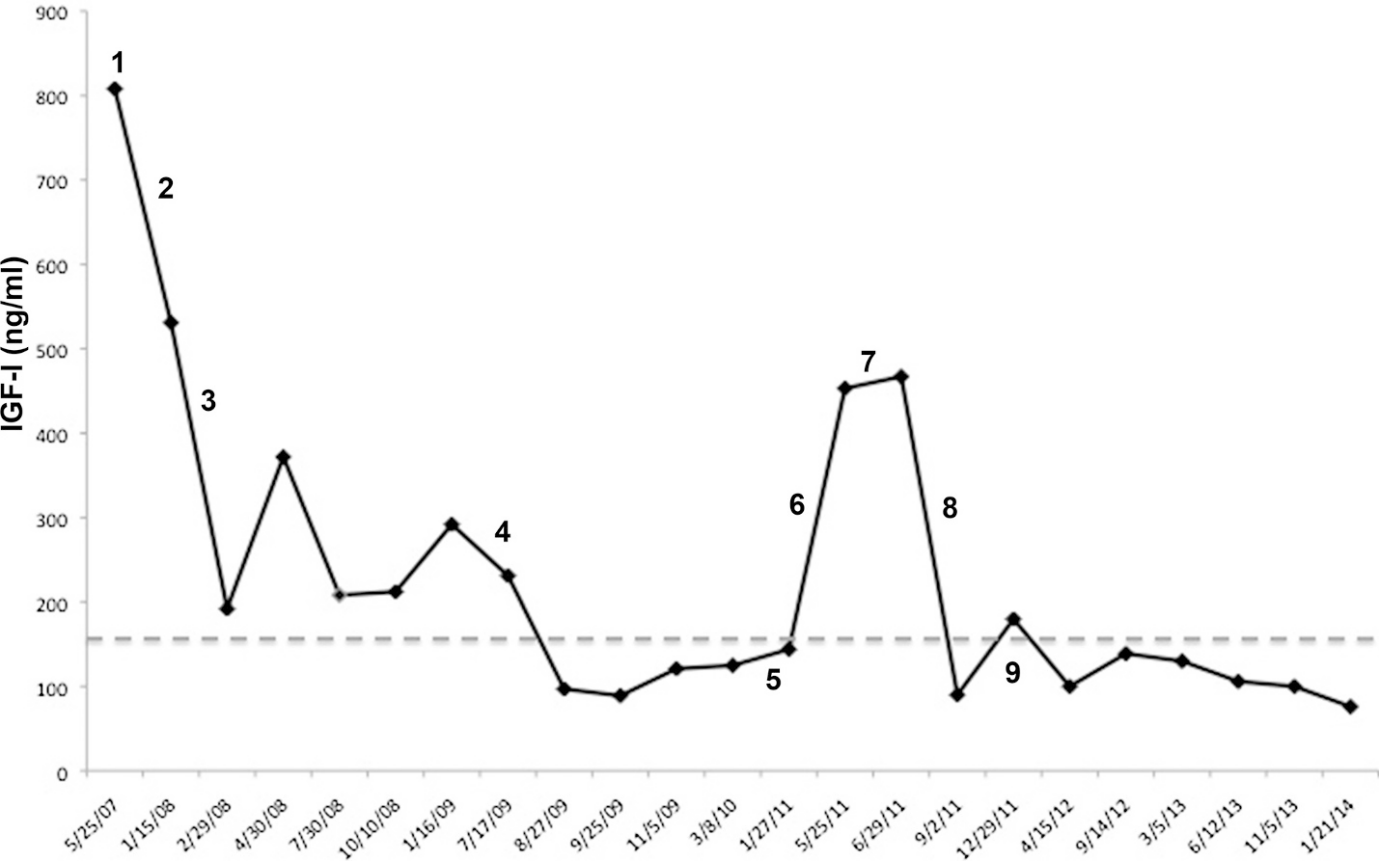
Serum levels of IGF-1 in relation to acromegaly control and breast cancer evolution. The dotted line represents the upper normal limit for IGF-I, 1. May 2007: Diagnosis of acromegaly (start of octreotide LAR treatment), 2. June 2007: Surgery for breast cancer, 3. February 2008: Trans-sphenoidal adenomectomy, 4. June 2009: Octreotide LAR withdrawal, start pegvisomant, 5. December 2010- January 2011: Diagnosis of lung metastasis of breast cancer, 6. February 2011: Start anastrazol and spontaneous pegvisomant withdrawal, 7. June 2011: Anastrazol withdrawal, start tamoxifen, 8. July 2011: Re-start pegvisomant, 9. September 2011: Reduction in diameter of pulmonary lesions

When first seen in our clinic, broad hands and feet were evident, which were reported to have increased in size over the years. Physical examination also revealed coarse facial features, enlargement of the nose, lips and ears, pronounced jaw with attendant macroglossia and teeth gapping, generalized thickening of the skin. Weight was 78 kg and height 172 cm, for a BMI of 26.36 kg/m2. Blood tests showed high levels of GH (85.9 ng/ml). Serum IGF-1 was much higher (770 ng/ml) than the expected elderly normal range (87–177 ng/ml). The serum levels of IGF-1 during the whole follow-up are illustrated in Fig. 1. The remaining pituitary hormones were within the normal range [Table 1] with the exception of a slightly elevated serum prolactin (30.8 μUI/ml. A diagnosis of active acromegaly was made. Pituitary MRI showed a macroadenoma (20 mm in maximum diameter). The sella turcica floor was depressed and the pituitary stalk was displaced. The patient refused surgery, and therapy with octreotide LAR (30 mg every 28 days) was started. Further diagnostic work-up revealed heart abnormalities (fibrosclerotic thickening of the mitral and aortic valve with mild aortic insufficiency) and prostate hypertrophy. Thyroid ultrasound confirmed a relapsing multinodular goiter (estimated thyroid volume: 89 ml). Fine needle aspiration cytology of the main thyroid nodules was consistent with benign lesions. Physical examination also revealed a firm nodule of the left breast (upper-outer quadrant) with palpable axillary nodes. Cytological examination of the breast lesion was diagnostic for malignancy. In June 2007, the patient underwent radical breast surgery. 12 lymph-nodes were surgically removed from the axillary region. At histology breast cancer was a hormone-sensitive (ER+ 70 %, PR+ 20 %), in situ ductal breast carcinoma with micro-infiltrating spots (<1 mm), (pT1mic, pN0M0 G2, Ki67 10 %, c-erb B2 DAKO HerceptTest 1+ neg). All examined lymph-nodes were free of metastatic involvement. The contralateral breast was evaluated by physical examination and ultrasound. A synchronous breast cancer was excluded. A detailed family history was also collected, which was negative for the presence of either breast or ovary cancer. The patient received post-surgical radiotherapy (50 Gy in the left mammary region) with no adjuvant systemic treatment.

**Table 1:** Hormonal findings at diagnosis of acromegaly

Parameters	Patient values	Normal values
GH (ng/ml)	85.9	<=1
IGF-I (ng/ml)	770	87–177
Prolactin (mcUI/ml)	30.8	2.5–17
ACTH (pg/ml)	33.3	0–71
Cortisol (mcg/dl)	16.7	5–23.3
TSH (mIU/l)	0.857	0.4–4
FT4 (ng/dL)	0.91	0.8–1.9
FT3 (pg/mL)	2.46	1.8–4.2
Tg Ab (IU/ml)	12.6	<60
TPO Ab (IU/ml)	13.7	<60
FSH (mIU/ml)	7.94	0.7–11.1
LH (mIu/ml)	2.67	0.8–7.6
Testosterone (ng/dl)	246	181–758

In February 2008, ten months after starting octreotide LAR, the serum levels of IGF-1 were still high (531 ng/ml) and the patient accepted to undergo trans-sphenoid surgery. Histological examination confirmed a somatothrope-cell pituitary adenoma. Immediately after pituitary surgery the IGF-1 serum levels decrease (192 ng/ml). Two months later, the IGF-1 serum levels raised again (372 ng/ml), which was consistent with the discovery of residual adenomatous tissue at pituitary MRI. Octreotide LAR treatment was re-instituted. 17 months after surgery, due to inadequate control of acromegaly on octreotide LAR (January 2009: GH = 7.5 ng/ml, IGF-1 = 292 ng/ml; July 2009: IGF-I = 231 ng/ml), pegvisomant (10 mg/day) treatment was started. Two months later, normal levels of serum IGF-I (97 ng/ml) were obtained.

**Fig. 2:**
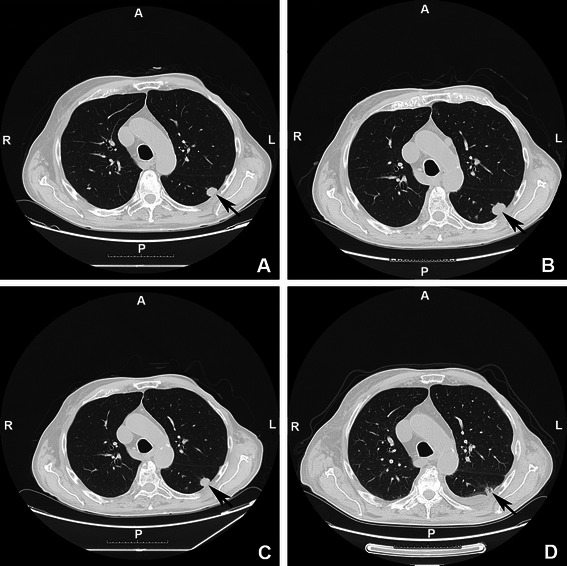
CT scan of the chest showing the radiological history of the metastatic lesion located in the apical segment of the lower lobe of the left lung. Panel **a** (01/2011): first evidence (maximum diameter 1.5 cm). Panel **b** (5/2011): dimensional increase after pegvisomant withdrawal (maximum diameter 2 cm). Panel **c** (09/2011): dimensional shrinkage after pegvisomant restart (maximum diameter 1.5 cm). Panel **d** (01/2014): further dimensional shrinkage under pegvisomant treatment (maximum diameter 1.1 cm)

In December 2010 a standard X-ray and a CT of the chest showed a metastatic lesion located in the lower lobe of the right lung, with a maximum diameter of 2.5 cm. A second metastatic lesion was evident in the apical segment of the lower lobe of the left lung (maximum diameter = 1.5 cm) [Fig. 2: Panel a]. The patient underwent right lower lobectomy. Histological examination confirmed the presence of a neoplastic nodule, which was diagnosed as metastatic breast cancer (ER+, Mammoglobin+). Hormone therapy with anastrozole (1 mg/day) was started. Meanwhile, contrary to the medical advice, the patient stopped pegvisomant treatment. IGF-1 serum levels increased again (453 ng/ml). In June 2011, progression of the metastatic lesion in the lower lobe of the left lung (from 1.5 cm to 2 cm maximum diameter) was observed at chest CT [Fig. 2: Panel b]. Two months later, a widespread metastatic disease of bone was detected at PET-FDG. Due to the neoplastic progression, the patient was shifted from Anastrazol to Tamoxifen (20 mg/day) treatment. In July 2011, the patient was re-started on pegvisomant treatment (10 mg/day), which led to a normalization of serum IGF-1 levels (90 ng/ml). A further chest CT was performed in September 2011 and displayed a significant reduction in size of the left lung metastasis (1.5 cm maximum diameter) [Fig. 2: Panel c]. In April 2012 this lung metastasis was found to be smaller than in the previous CT (1.1 cm of maximum diameter). Subsequent CT scans, performed 6, 12 and 20 months later [Fig. 2: Panel d], showed no progression of the metastatic lung disease. The patient remains in fair clinical conditions, has a good control of acromegaly (January 2014: IGF-1 = 76 ng/ml) and his treatment still includes pegvisomant for acromegaly and tamoxifen for metastatic breast cancer.

## Conclusions

We report the case of a caucasian male patient who initially suffered from colorectal cancer and subsequently was diagnosed with acromegaly and breast cancer. Acromegaly was not cured by trans-sphenoidal surgery, was partially resistant to octreotide LAR and was successfully controlled by pegvisomant treatment, as assessed by IGF-1 serum measurements. Clinically relevant changes in the progression of breast cancer and metastatic lesions were observed in relation to the metabolic control of acromegaly. A few peculiar aspects deserve discussion.

First, the patient was a man and, to the best of our knowledge, there is no previous description of a male patient with acromegaly and breast cancer. Even in the largest series of male patients with breast cancer [13], the Authors did not report a case associated with acromegaly. Although a single observation does not allow establishing a causal link between the two pathologic conditions, the fact that both acromegaly (40–90 cases/million/year) and male breast cancer (10 cases/million/year) are rare diseases argues against a chance association. Although the mechanisms by which acromegaly results in an increased risk of cancer have not been fully clarified, in vitro studies demonstrated that GH and IGF-1 have a proliferative and anti-apoptotic effect on different cell lines, eventually leading to neoplastic evolution [14]. Moreover, high serum levels of GH and IGF-1 enhance the proliferation rate of epithelial cells of the sigma crypts, which, by increasing the length of colon and sigma, would predispose to the development of colon-rectal cancer [15]. IGF-1, after binding to its receptor, is also known to increase the proliferation rate of estrogen-dependent breast cancer cell lines. Based on the above evidence, the co-existence of breast cancer and acromegaly might not be surprising. Given the long history of acromegaly, it also conceivable to believe that in our patient a cause effect relationship could exist between the endocrine disease and colon-rectal cancer. A genetic background linking GH secreting pituitary adenomas, colon-rectal cancer and breast cancer might also be hypothesized. In a study by Georgitsi et al., the Authors evaluated the role of AIP mutations, which are known to occur in pituitary adenomas, in the genesis of common cancers (prostate, breast and colon-rectal cancer). However, no AIP mutation could be detected in these types of cancer. To our knowledge, no other study evaluated the existence of genetic link between acromegaly, colon-rectal and breast cancer [16].

Second, the aggressive behavior of breast cancer was unexpected in this patient, due to the favorable prognostic TNM stage at diagnosis (pT1a mic, N0). Also intriguing is the observation that the metastatic progression of breast cancer was temporally related to the interruption of pegvisomant treatment. Due to the lack of proper investigations, we cannot make any speculation about the association between acromegaly control and the onset of breast cancer metastasis. However, on the other hand, the rapid spread of the metastatic lesions occurred concomitantly to a rise in serum IGF-1 levels. The subsequent normalization of IGF-1, due to the re-introduction of pegvisomant treatment, was followed by a reduction in size and a stabilization of the lung metastasis. This sequence of events is in agreement with the observations of Chae et al. [17], who reported an impressive amelioration of metastatic breast cancer in a female patient with acromegaly being treated with octreotide LAR. At variance with octreotide LAR, in vivo data on the anti-cancer effect of pegvisomant are scanty. Yin et al. demonstrated that pegvisomant is more effective than octreotide LAR in suppressing the GH-IGF-1 axis, thus implying that this GH-receptor blocker drug could be effective in treating several types of tumors, such as breast and colon-rectal cancers [18].

The apparent failure of the elective treatment with anastrozole and the favorable response to tamoxifen could be explained by several factors. Previous studies demonstrated that cross talks between the signaling pathways of estrogen receptor (ER) and IGF-1 receptor (R) play a crucial role in determining resistance to anti-estrogen endocrine therapy in breast cancer [19–21]. In vitro experiments by Lisztwan et al. demonstrated that adding the aromatase blocker letrozole to an inhibitor of the IGF-1 R pathway synergistically blocks the proliferation and induces apoptosis of breast cancer cells [22]. Taken together, these findings would suggest that a dual targeting of IGF-1 R and ER pathways would prevent or delay the onset of endocrine resistance. At variance with this assumption, a randomized, open-label, phase III trial reported similar event-free, recurrence-free, and overall survival in 667 postmenopausal women with ER-positive early-stage breast cancer who received treatment with tamoxifen or tamoxifen + octreotide LAR as adjuvant therapy [23]. However, these negative findings were obtained in patients with normal GH and IGF-1, a completely different clinical situation from that of our acromegalic patient who, due to the high levels of circulating IGF-1, had a strong activation of the IGF-1-R pathway. In turn, this IGF-1 R activation could explain the apparent resistance to anastrozole and the benefits observed with tamoxifen. Indeed, anastrozole was administered when the circulating levels of IGF-1 were high due to the interruption of pegvisomant treatment, while the shift to tamoxifen coincided with re-starting pegvisomant and the subsequent metabolic control of acromegaly. Moreover, tamoxifen itself might have been responsible for a further reduction of IGF-1 serum levels. This IGF-1 lowering effect of tamoxifen was previously described both in acromegalic patients and in healthy subjects [24–27].

In conclusion, we report for the first time the case of a male acromegalic patient with breast cancer. The clinical evolution of breast cancer in our patient highlights the concept that the control of acromegaly might play a role in the outcome of the coexistent neoplasia. No matter how the control of acromegaly is achieved, suppression of IGF-1 should be mandatory in acromegalic patients with cancer.

### Consent

Written informed consent was obtained from the patient for publication of this Case report and any accompanying images. A copy of the written consent is available for review by the Editor of this journal.
